# Reflecting on Equity in Perinatal Care During a Pandemic

**DOI:** 10.1089/heq.2020.0022

**Published:** 2020-07-28

**Authors:** P. Mimi Niles, Ifeyinwa V. Asiodu, Joia Crear-Perry, Zoë Julian, Audrey Lyndon, Monica R. McLemore, Arianna M. Planey, Karen A. Scott, Saraswathi Vedam

**Affiliations:** ^1^ Department of Family Practice, Midwifery Program, University of British Columbia, Vancouver, Canada.; ^2^ School of Nursing, University of California, San Francisco, California, USA.; ^3^ National Birth Equity Collaborative, New Orleans, Louisiana, USA.; ^4^ Department of Obstetrics, Gynecology and Reproductive Sciences, University of California, San Francisco, California, USA.; ^5^ Rory Meyers College of Nursing, New York University, New York, New York, USA.; ^6^ Geography and Geographic Information Science, University of Illinois, Urbana, Illinois, USA.

**Keywords:** health disparities, public health, reproductive health, minority health

## Abstract

Growing discourse around maternity care during the pandemic offers an opportunity to reflect on how this crisis has amplified inequities in health care. We argue that policies upholding the rights of birthing people, and policies decreasing the risk of COVID-19 transmission are not mutually exclusive. The explicit lack of standardization of evidence-based maternity care, whether expressed in clinical protocols or institutional policy, has disproportionately impacted marginalized communities. If these factors remain unexamined, then it would seem that equity is not the priority, but retaining power and control is. We advocate for a comprehensive understanding of how this pandemic has revealed our deepest failures.

Growing discourse around maternity care during the pandemic amplifies long-standing disparities and inequities in health care. The same communities that are being devastated by COVID-19 have long suffered poor maternal health care outcomes.^1^ In the United States, even when accounting for socioeconomic and education factors, black and indigenous people are 2–4 times more likely to die from a pregnancy-related complication irrespective of income and education.^2^ Deeply seeded racist policies and practices alongside a lack of robust well-funded social services, in an anemic public health care system, serve as the causal agents of disparities.^3^

A more robust critique of the health care responsiveness to the pandemic must include thoughtful analysis of why systems were not put in place to protect both patients' and providers' well-being, while simultaneously preserving dignity in maternity care. It must also include an acceptance that the lack of standardization of evidence-based maternity care, whether expressed in clinical protocols or institutional policy, perpetually fails the people we care for and acutely impacts historically disenfranchised people.

As one example, in response to the pandemic, some New York City hospitals, rapidly created policies, barred partners from the labor rooms, leaving women to labor and birth alone. These policies generated a groundswell of support advocating for the presence of supportive partners as being critical to the safety and well-being of women, which led to an executive order signed by Governor Cuomo prohibiting hospitals from enforcing this restrictive policy.^4^ In response, some have suggested that a policy that allows the presence of support partners is “uninformed and unethical” and places undue strain on providers who lack personal protective equipment (PPE), potentiating the spread of the virus,^5^ thereby creating a rivalrous division. We, a group of health care providers, researchers and advocates, fundamentally uphold the rights of providers and patients. We suggest that creating and perpetuating a tension or a false choice between choosing providers' rights over patients' rights further alienates patients from their own care. In addition, limiting the discussion to the lack of PPE is reductionistic and limits an equity-based understanding of where the system has failed to secure safety for *both* providers and patients.

We conducted a thoughtful review of the available literature regarding COVID-19, in the framework of existing literature on equitable maternity care that centers the lived experiences of birthing people. We disrupt the myth that policies that uphold the human rights of birthing people and policies that decrease risk of COVID-19 transmission are mutually exclusive. We submit three main contributors to perpetuating inequitable maternity care:

(1) The lack of rigor in using case studies to suggest a change in evidence-based practice.
(2) The lack of evidence-based solutions that address the root causes of inequitable access to services, resources, and equipment.
(3) The continued failure to apply a comprehensive human rights-based reproductive justice lens to how we care for birthing people and families.

To date, research about maternity care during COVID-19 has been restricted to small case studies, which is warranted as little is known about this virus. However, citing case studies as an indication to change protocols is poor science and unnecessarily contributes to fear among both parents and providers. A blog post by two physicians references a local news report of a neonatal death of 7-week-old infant in Connecticut to suggest possible evidence of “vertical transmission.” However, most case studies out of Korea, China, Italy, and the United States show that all neonates tested negative, providing further data that vertical transmission is unlikely.^6,7^ Physician in NY also reported two cases of acute decompensation for two patients with COVID-19 who required intubation.^8^ Both people underwent cesareans, which lengthened their exposure, due to increased length of stay and increased and intensive contact with providers. Neither case report states how long they were in the hospital nor their repeated exposure to the overall health care system leading up to their inductions. Although most case reports caution about exposure risk to providers, they fail to mention iatrogenic risk to patients who have repeated and prolonged engagement with providers and hospital facilities that are the hot spots for infections. However, implementing practice changes based on scant evidence, while ignoring established best practice guidelines alongside limiting patient choice and autonomy, suggests that equitable respectful care will be the first principles to be sacrificed in a crisis.

Professional organizations, the American College of Obstetricians and Gynecologists (ACOG) or the Society for Maternal Fetal Medicine (SMFM), have yet to address or understand the potential long-term health impacts of the overuse of interventions during this pandemic.^9^ Advocacy and guidelines around reducing the use of interventions that require multiple care interactions over a longer course of stay are lacking. Although the frequency of prenatal and postpartum visits has been reduced^10^ early reports of increased medical interventions serve to increase close contact and exposure for both patients and providers. For example, recommending early inductions and elective cesareans to manage hospital census and staffing during COVID-19 actually means that patients will require more intensive contact with numerous admitting staff, nurses, and physicians, and thus greater exposure to both COVID-19 and iatrogenic pathogens in the hospital environment. These interventions also link to longer length of inpatient stays, more invasive interventions, and the subsequent increase in risks of obstetric complications. Given that women of color already experiences higher rates of inductions and cesareans, these policies are likely further exacerbate the disparities in outcomes.^11,12^ We suggest that pandemic data should be carefully analyzed, particularly in the context of lower thresholds for interventions and the potential impact on increased inductions and cesarean births, even though SARS CoV-2 is not an indication for either. The same logic around decreasing intensive contact should be applied to labor and birth practices during the pandemic.

Conversely, it is well established that the most important resource to ensure safe birth is the constant presence of a labor support companion.^13,14^ When a partner is present they can and will provide the repeated close and contact heavy labor support measures over hours, thereby allowing nurses to remain more socially distant. The World Health Organization has offered a clear evidence-based recommendation that all laboring persons have a support person present.^15,16^ When a known support person is present, they can provide needed labor support that involves close contact and physical touch, thereby reducing repeated exposure for providers. A policy of no support persons unduly impacts marginalized communities and implicitly reinforces the “sacrificial” or expendable status of Black and indigenous parents, who have long borne the consequences of mistreatment and abandonment in their health care experiences.^17,18^ Before the pandemic, these communities disproportionately carry the burden of unequal treatment, with high rate of cesareans, higher rates of preterm births, and higher overall rates of maternal morbidities and mortalities.^19–21^ An overzealous reaction that only ensures short-term health, with scant commitment to the long-term health consequences, creates poorer outcomes in the future for Black, Latinx, indigenous, and other marginalized groups.

Weaponizing fear among pregnant people and providers as a means to “assure” safe births, with little consideration of the long-term sequelae to these actions, generates greater harm and fear. Those who advocate for keeping partners out of the birth settings as the principal means of protecting the safety of providers or for separating mothers from their infants indicate a failure to examine the system level inequities that also impact the care management decisions made during a pandemic. As a result, we predict that an uptick in “too much, too soon” approach will be the defining critique of how maternity care systems and providers responded to the pandemic, whereas the “too little, too late” will be how principles of respect and dignity in care were not universally applied.^22^ If these factors remain unexamined within the context of what we know is killing black people and other marginalized people in the perinatal period—then it would seem that safety is not actually the priority, but retaining power and control are. And if we are to truly claim the banner of “supporting reproductive rights” and providing “patient-centered care” then we must be even more unwavering in our commitment to operationalizing these care frameworks during times of crisis.^23^ Imagining and creating a responsive and compassionate systems of care that centers the most marginalized parents and families among us must be our defining legacy.

## Abbreviations Used

ACOG: American College of Obstetricians and Gynecologists
PPE: personal protective equipment
SMFM: Society for Maternal Fetal Medicine
